# Transthyretin Upregulates Long Non-Coding RNA MEG3 by Affecting PABPC1 in Diabetic Retinopathy

**DOI:** 10.3390/ijms20246313

**Published:** 2019-12-13

**Authors:** Guangming Fan, Yu Gu, Jiaojiao Zhang, Yu Xin, Jun Shao, Francesca Giampieri, Maurizio Battino

**Affiliations:** 1 Key Laboratory of Industry Biotechnology, Ministry of Education, School of Biotechnology, Jiangnan University, Wuxi 214122, China; m17851315181@163.com (G.F.); guyu96116@126.com (Y.G.); 2 Department of Clinical Sciences, Faculty of Medicine, Università Politecnica delle Marche, 60131 Ancona, Italy; zh.jojo@yahoo.com (J.Z.); f.giampieri@univpm.it (F.G.); 3 Nutrition and Food Science Group, Department of Analytical and Food Chemistry, CITACA, CACTI, University of Vigo—Vigo Campus, 36201 Vigo, Spain; 4 International Research Center for Food Nutrition and Safety, Jiangsu University, Zhenjiang 212013, China

**Keywords:** diabetic retinopathy (DR), transthyretin (TTR), long non-coding RNA (lncRNA), *maternally expressed gene 3* (MEG3), polyadenylate-binding protein cytoplasmic 1 (PABPC1)

## Abstract

The aim of the study was to demonstrate how transthyretin (TTR) could affect long non-coding RNA (lncRNA) of maternally expressed gene 3 (MEG3) and play important roles in diabetic retinopathy (DR). A DR model in C57BL/6 mice was established after intraperitoneal injection of streptozotocin (STZ). After intravitreal injection with TTR pAAV vector, MEG3 short hairpin RNA (shRNA), scrambled shRNA, or MEG3, retinal imaging, retinal trypsin digestion, and fundus vascular permeability tests were performed. Cell counting kit-8 (CCK8), transwell, and Matrigel assays were employed to detect the proliferation and migration of human retinal microvascular endothelial cells (hRECs). The binding between long non-coding RNA of maternally expressed gene 3 (lncRNA-MEG3) and microRNA-223-3p (miR-223-3p) was observed by using luciferase reporter assays, while co-immunoprecipitation (co-IP) was employed to confirm the interaction between TTR and the target. In the DR mice model, retinal vascular leakage and angiogenesis were repressed by overexpressing TTR. In vitro, the added TTR promoted the level of lncRNA-MEG3 by interacting with poly (A) binding protein cytoplasmic 1 (PABPC1), and then repressed proliferation and angiogenesis of hRECs. In vivo, silencing or overexpressing lncRNA-MEG3 significantly affected retinal vascular phenotypes. Additionally, the interaction between lncRNA-MEG3 and miR-223-3p was confirmed, and silencing of miR-223-3p revealed similar effects on hRECs as overexpression of lncRNA-MEG3. In summary, in the DR environment, TTR might affect the lncRNA MEG3/miR-223-3p axis by the direct binding with PABPC1, and finally repress retinal vessel proliferation.

## 1. Introduction

Diabetic retinopathy (DR) is considered to be among the most severe causes of vision impairment and loss in the working-aged and elderly population [1], and the incidence of DR increases annually worldwide [2,3,4,5]. Due to the complex etiology of DR and other factors, the pathogenesis of DR is not entirely clear [6]. Therefore, continuing research is necessary to elucidate the pathogenesis and underlying molecular mechanisms of the development and progression of DR.

In the eyes, transthyretin (TTR) is mainly expressed in human retinal pigment epithelial cells (hRPECs) and the choroid [7], and it usually works as the carrier of thyroxine (T4) and retinol [8,9]. As previously reported, TTR should be correlated with diabetes-associated diseases, e.g., type I diabetes patients showed lower serum TTR levels [10]. In clinical investigations, myopia was revealed to protect diabetic patients from suffering DR [11,12]. Our previous work has demonstrated that higher vitreous TTR content of high myopia patient [13] might help to prevent the progression of DR [14]. The serum and vitreous TTR levels in DR patients were associated with DR progression [15]; TTR suppressed angiogenesis by affecting the angiopoietin-Tie signaling pathway in hyperglycemia [16], and enhanced the apoptosis of hRECs through a hypoxia-associated 78-kDa glucose-regulated protein (GRP78)-dependent pathway [17]. Still, the regulatory mechanisms involving TTR in DR are not entirely clear.

Long non-coding RNAs (lncRNAs) regulate targeted mRNA expression via the microRNA (miRNA) response element known as competing endogenous RNA (ceRNA) [18,19]. LncRNAs are known to play vital roles in ocular disease [20], including glaucoma [21,22], retinoblastoma [23,24], and DR [25,26]. Recently, the study of lncRNAs in DR has become a hot point. lncRNAs of RNCR2, NEAT2, CDKN2B-AS1, and PVT1 have shown significant diagnostic performance in DR progression [27], and lncRNA-MALAT1 promotes neovascularization in DR through regulating the miR-125b/VE-cadherin axis [28]. lncRNA H19 prevents endothelial–mesenchymal transition in DR [29].

The long non-coding RNA of maternally expressed gene 3 (lncRNA-MEG3)/miR223/NLRP3 inflammasome gene axis is thought to play a significant role in pyroptosis of endothelial cells [30]. The decrease in lncRNA-MEG3 could enhance retinal vessel dysfunction through the PI3k/Akt signaling pathway [31]. In our previous work using miRNA microarray and qRT-PCR assays, miR223-3p was upregulated in serum and aqueous humor of DR patients, and TTR was proved to affect neovascularization in DR through the STAT4/miR-223-3p/FBXW7 signaling pathway [32]. However, how lncRNA-MEG3 interacts with TTR in DR remains to be explored.

As the interaction between lncRNA-MEG3 and miR223-3p has been reported in human aortic endothelial cells (HAECs) [30], in the current study we aim to investigate: (1) the potential relationship between TTR and lncRNA-MEG3; and (2) the relationship between lncRNA-MEG3 and poly(A) binding protein cytoplasmic 1 (PABPC1), on the basis that the co-immunoprecipitator, PABPC1, has been identified as the direct binding target of TTR and has been reported to bind the poly (A) tails of mRNAs, regulating the stability and biofunction of lncRNAs [33,34,35],.

This study was designed to elucidate the details of the interactions between TTR and miR223-3p, including the potential direct targets of TTR (PABPC1) and miR223-3p (lncRNA-MEG3) in DR, both in vivo and in vitro, which might provide new principles on the molecular pathogenesis, clinical prevention, and therapy of DR.

## 2. Results

### 2.1. The Protective Effects of TTR on Retinas of DR Mice

**Figure 1:**
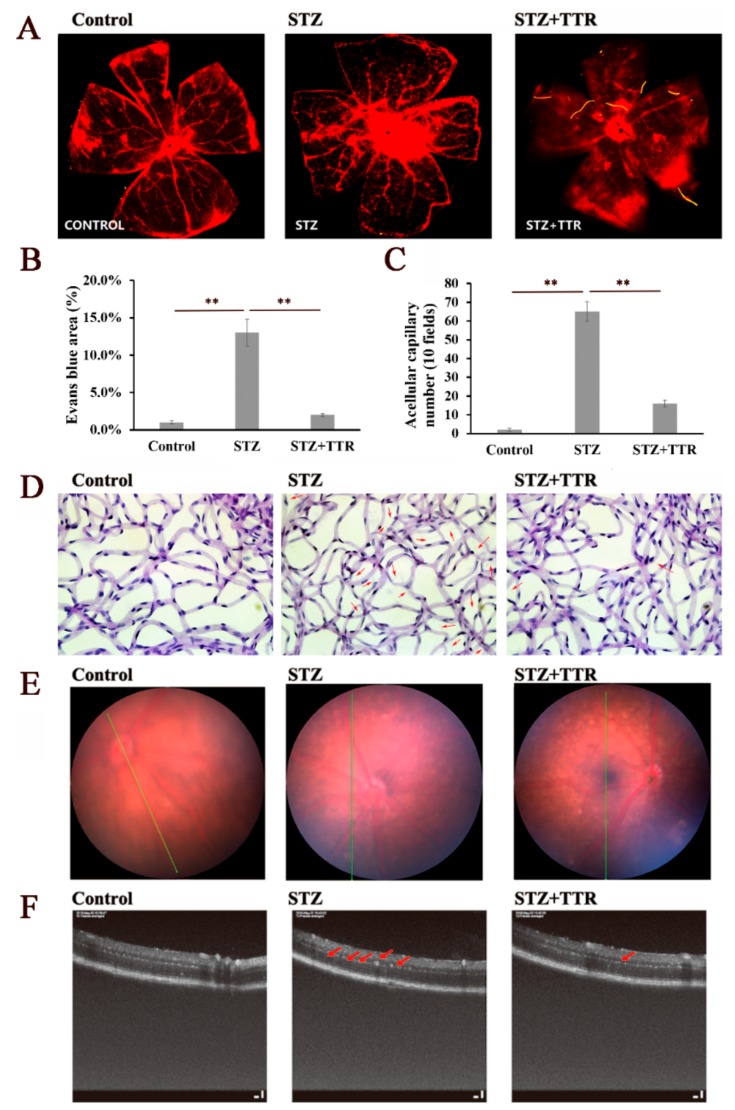
TTR regulates diabetes mellitus-induced retinal angiogenesis in vivo. (**A**,**B**) The retinas of control (*n* = 6), STZ (*n* = 3), and STZ + TTR (*n* = 4) mice infused with Evans blue dye for 2 h were stripped and scanned using a fluorescence microscope. The severe retinal vascular leakage of STZ-induced diabetic retinopathy (DR) mice could be reversed with the overexpressed TTR (** *p* < 0.01) (×100 magnification times). (**C**,**D**) The retinas of control (*n* = 6), STZ (*n* = 3), and STZ + TTR (*n* = 4) mice were applied for trypsin digestion; the acellular retinas of STZ-induced DR mice revealed a significantly higher capillary number (quantified in 10 fields per retina, pointed with red arrows), while overexpressed TTR showed the reverse of this phenomenon (** *p* < 0.01) (×400 magnification times). (**E**) Spectral domain fundal photographs of normal, STZ, and TTR mice; there was no significant difference (×100 magnification times). (**F**) Spectral domain optical coherence tomography (OCT) imaging of normal, STZ, and TTR mice, and the hard exudate of the retina is marked with red arrows (×400 magnification times).

The progression of DR was characterized by abnormal retinal microvasculature, reduced retinal perfusion, increased vascular permeability, and pathological intraocular proliferation of retinal vessels. Diabetic mice were induced with intraperitoneal injection of streptozotocin (STZ), and after intravitreal injection with TTR pAAV vector, MEG3 short hairpin RNA (shRNA), scrambled shRNA, or MEG3, the serum glucose level and weight of these mice revealed no significant fluctuations after three months (Table 1). As evaluated by the Evans Blue (EB) leakage assay, overexpressed TTR repressed diabetes-associated retinal vascular leakage (Figure 1A,B), and the retinal trypsin digestion assay demonstrated that overexpressed TTR partially reversed vascular hyperglycemia-induced pericyte loss and aggravated capillary degeneration (Figure 1C,D). In addition, the results of the retinal optical tomography scan and fundus photography assays exhibited significantly reduced hard exudate in diabetic mice. Reduced overexpression of TTR resulted in changes that could be observed in DR, partially reversing vascular distortion (Figure 1E,F).

### 2.2. TTR Interacted with Poly(A) Binding Protein Cytoplasmic 1 (PABPC1) to Stabilize lncRNA-MEG3

**Figure 2:**
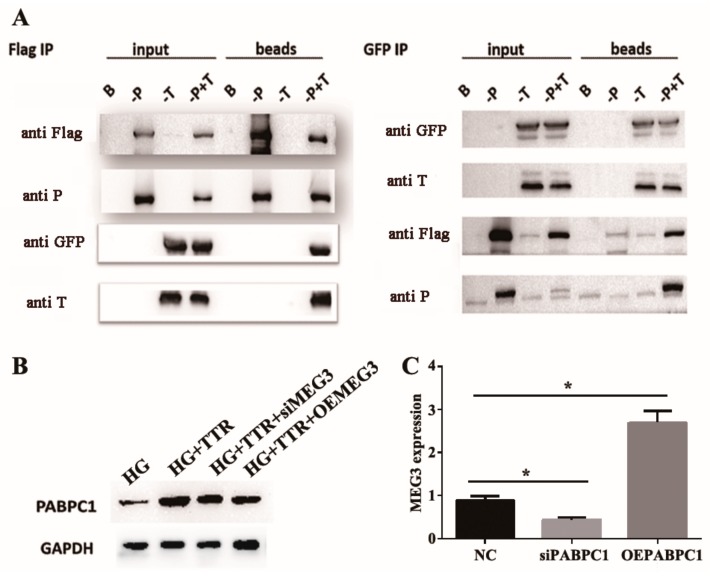
Poly (A) binding protein cytoplasmic 1 (PABPC1) interacts with long non-coding RNA of MEG3 (lncRNA-MEG3) and increases the stability of lncRNA-MEG3. (**A**) The bind of TTR and PABPC1 was confirmed by co-immunoprecipitation (co-IP). PABPC1 and TTR were expressed or co-expressed in HEK293 cells; PABPC1 and TTR were fused with Flag- and GFP-tags, respectively. B indicates blank (IgG), -P indicates PABPC1, -T indicates TTR, and -P + T represents co-expressed PABPC1 and TTR. Input samples were used as controls without Protein A beads. Protein A beads-Anti Flag IgG was used to capture PABPC1 or PABPC1-TTR complex, while Protein A beads-Anti GFP IgG was used to capture TTR or the TTR-PABPC1 complex. (**B**) The levels of PABPC1 was tested by western blot in HG, TTR, and TTR with overexpression of lncRNA-MEG3 (OE lncRNA-MEG3) or small interfering lncRNA-MEG3 (silncRNA-MEG3). (**C**) The relationship between lncRNA-MEG3 and PABPC1 was tested by qRT-PCR and this experiment was repeated three times (* *p* < 0.05).

Co-immunoprecipitation (Co-IP) assays in human embryonic kidney 293 (HEK293) cells confirmed the interaction between TTR and PABPC1 (Figure 2A); in hRECs, western blot analysis indicated that PABPC1 levels increased with TTR, while lncRNA-MEG3 overexpression or knockdown showed no significant effects on the PABPC1 level with TTR (Figure 2B) in high-glucose environments. Previous studies demonstrated that PABPC1 participates in regulation pathways and lncRNA stabilization [33,34,35]. To investigate whether PABPC1 regulated the stability of lncRNA-MEG3, actinomycin D was used to inhibit RNA synthesis in hRECs. PABPC1 knockdown significantly and rapidly decreased the level of lncRNA-MEG3, while overexpression of PABPC1 caused the reverse (Figure 2C). These results indicated that TTR enhances PABPC1 content by interacting with PABPC1 directly, and could even further stabilize lncRNA-MEG3 under in vitro hyperglycemic conditions.

### 2.3. TTR Effects Proliferation, Migration, and Vascularization by Regulating lncRNA-MEG3

**Figure 3:**
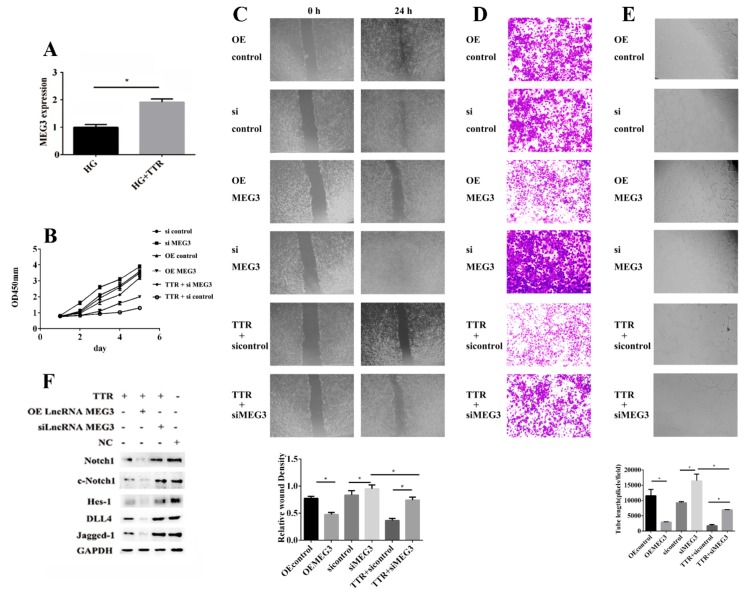
TTR effects proliferation, migration and vascularization by regulating lncRNA-MEG3. (**A**) In qRT-PCR, the expression level of MEG3 could be enhanced by TTR (repeated independently three times; *, *p* < 0.05). (**B**) Cell viability was determined using cell counting kit-8 (CCK8) assay; TTR or overexpression of MEG3 (OEMEG3) could inhibit the proliferation of human retinal microvascular endothelial cells (hRECs), while knockdown of MEG3 (siMEG3) could reverse the inhibition function of TTR (repeated independently three times). (**C**) In wound healing analysis, overexpression of MEG3 or the addition of exogenous TTR could significantly repress the wound healing process, while knockdown of MEG3 showed the opposite phenomenon (repeated independently three times; *, *p* < 0.05) (×100 magnification times). (**D**) In transwell assay, overexpression of MEG3 or the addition of exogenous TTR could significantly repress cell migration, while knockdown of MEG3 enhanced the migration trend (*, *p* < 0.05) (×100 magnification times). (**E**) In tube formation assay, tube-like structures were observed 6 h after cell seeding. Average length of tube formation for each field was statistically analyzed for at least three independent experiments. Overexpression of MEG3 or the addition of exogenous TTR could significantly suppress tube formation process of hRECs, while knockdown of MEG3 enhanced it (*, *p* < 0.05) (×100 magnification times). (**F**) In western blot analysis, TTR could regulate the levels of key proteins in Notch pathway; overexpression of MEG3 could enhance this trend, while knockdown of MEG3 could partially rescue it.

Our previous work has exhibited that TTR inhibits retinal vascular proliferation [14,15,16,17]. In this current work, lncRNA-MEG3 levels were increased by adding TTR (Figure 3A). After hRECs were treated with pcDNA3.1-OEMEG3, pcDNA3.1-siMEG3, pcDNA3.1-siMEG3, and TTR, or treated with pcDNA3.1-sicontrol and TTR, cell counting kit-8 (CCK8) analysis revealed that lncRNA-MEG3 overexpression inhibited hREC proliferation, while lncRNA-MEG3 knockdown reversed TTR inhibition of hREC proliferation (Figure 3B). The wound healing, transwell, and tube formation assays demonstrated that during lncRNA-MEG3 knockdown, TTR did not significantly affect hREC proliferation, migration, and vascularization, and lncRNA-MEG3 overexpression enhanced TTR inhibition of hRECs (Figure 3C–E).

The Notch signal pathway plays vital roles in cell proliferation [36], and miR-223-3p has been proved to promote the level of Notch1 in hRECs in our previous work [32]. In order to find out whether the level of MEG3 could act on this pathway, western blot analysis was employed; the results showed that TTR treatment suppressed the levels of key factors in this pathway including Notch1, c-Notch1 (cleaved Notch1), Jagged-1, Delat-like protein 4 (DLL4), and Hes-1; lncRNA-MEG3 overexpression enhanced this trend, while lncRNA-MEG3 knockdown partially rescued this phenomenon (Figure 3F). These data suggest that TTR could inhibit neovascularization by upregulating the level of lncRNA-MEG3.

**Figure 4:**
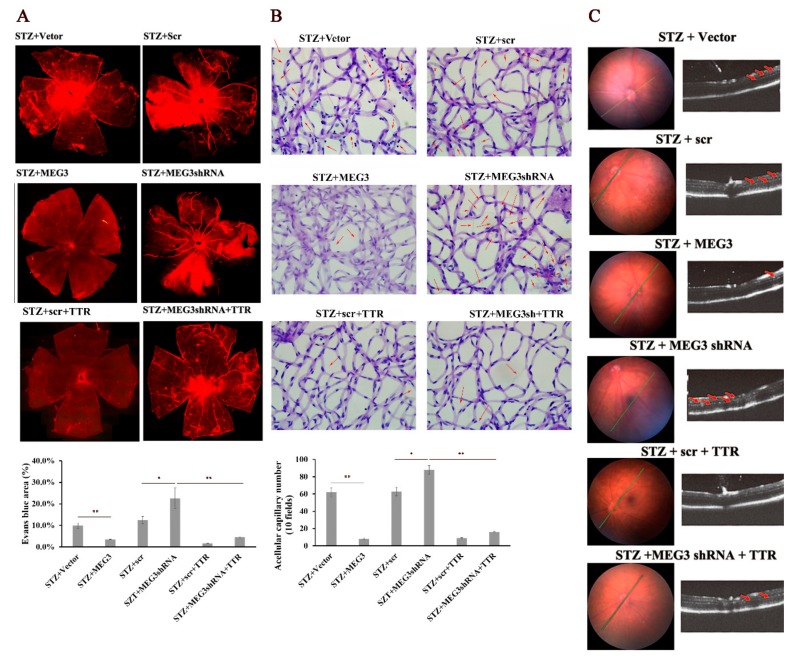
TTR regulates diabetes mellitus-induced retinal angiogenesis by MEG3 in vivo. (**A**) The retinas of STZ + vector (*n* = 3), STZ + scr (*n* = 3), STZ + MEG3 (*n* = 3), STZ + MEG3 shRNA (*n* = 3), STZ + scr + TTR (*n* = 3), and STZ + MEG3 shRNA + TTR (*n* = 4) mice infused with Evans blue dye for 2 h were stripped and observed using a fluorescence microscope. Overexpression of MEG3 (OEMEG3) and TTR could significantly decrease the retinal vascular leakage of STZ-induced DR mice, and knockdown of MEG3 with MEG3shRNA could promote retinal vascular leakage (*, *p* < 0.05; **, *p* < 0.01) (×100 magnification times). (**B**) The retinas of STZ + vector (*n* = 4), STZ + scr (*n* = 3), STZ + MEG3 (*n* = 4), STZ + MEG3 shRNA (*n* = 3), STZ + scr + TTR (*n* = 4), and STZ + MEG3 shRNA + TTR (*n* = 4) mice were applied for trypsin digestion. Overexpression of MEG3 and TTR could reduce the capillary number in acellular retinas of STZ-induced DR mice (quantified in 30 fields per retina and average, signaled with red arrows), and knockdown of MEG3 showed an opposite function, using an empty vector and scr as blanks (*, *p* < 0.05; **, *p* < 0.01) (×400 magnification times). (**C**) Spectral domain fundal photographs showed no significant difference. In spectral domain optical coherence tomography (OCT) imaging of mice, the hard exudate of retina was marked with red arrows; overexpression of MEG3 and TTR could reduce hard exudate of retina, but knockdown of MEG3 enhanced it. scr: scrambled short-hairpin RNA.

In vivo, overexpression of lncRNA-MEG3 educed retinal hard exudates, cellular capillary number, and retinal vascular leakage, while lncRNA-MEG3 shRNA had the opposite effects. Further, TTR reversed the retinal dysfunction resulting from lncRNA-MEG3 shRNA (Figure 4A–C). These data indicate that TTR might affect DR neovascularization through lncRNA-MEG3.

### 2.4. LncRNA-MEG3 Functions as the ceRNA of miR-223-3p in DR

**Figure 5:**
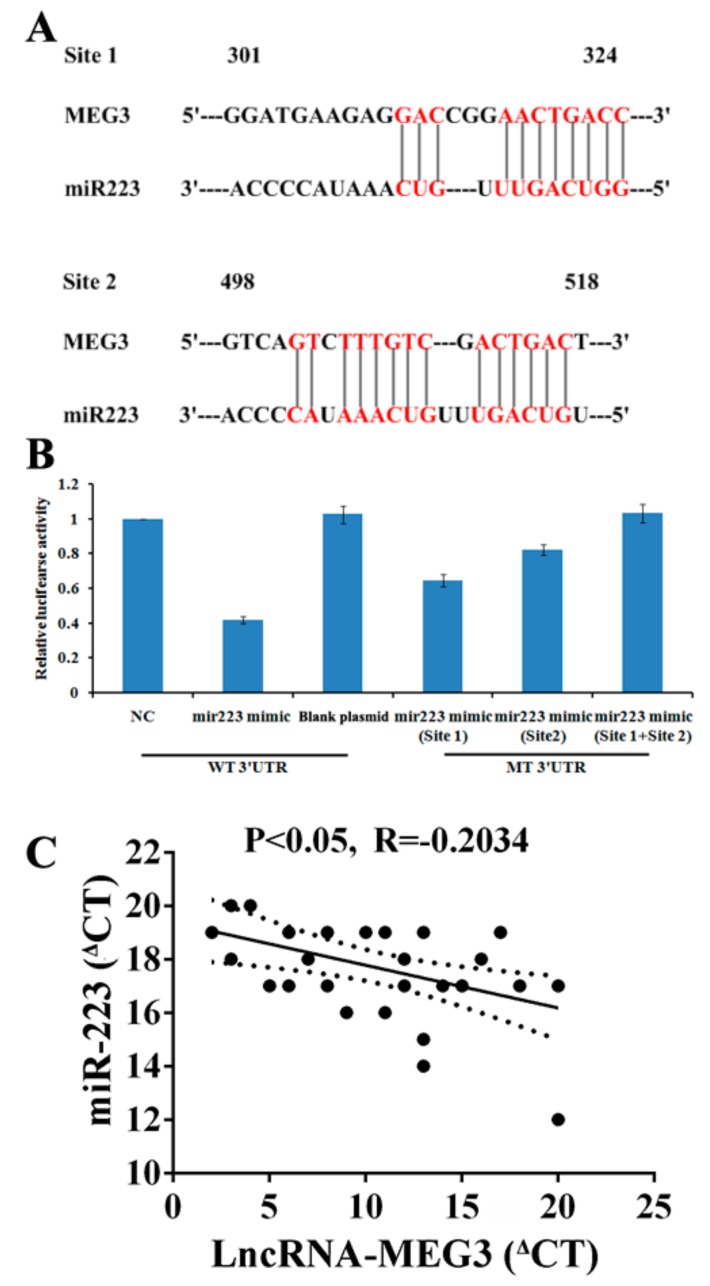
LncRNA-MEG3 works as a miR-223-3p sponge in hRECs. Identification of lncRNA-MEG3 as a competing endogenous RNA (ceRNA) for miR-223-3p. (**A**) Sequence complementarity between MEG3 and miR-223-3p. The letters in red indicate matched bases. (**B**) Luciferase reporter constructs containing pcDNA3.1-MEG3 plasmid with the full-length MEG3 sequence. Luciferase activities with wild-type pcDNA3.1- MEG3 plasmid (LncRNA- MEG3) or mutant (mut) sequences of MEG3 at the two binding sites for miR-223-3p, site 1 (mutant 301- 324 gene locus of MEG3) and site 2 (mutant 498- 518 gene locus of MEG3), or both. The experiment was repeated independently three times. (**C**) The correlation between the transcript levels of lncRNA-MEG3 and miR-223-3p was measured in hRECs. The ^Δ^CT values were subjected to Pearson correlation analysis.

As previously reported, lncRNAs could competitively bind common miRNAs as ceRNAs [25,37,38] and regulate miR-223-3p expression by lncRNA-MEG3, which has been observed in other diseases [30]. In this study, “RegRNA” and “MiRCode” were used to predict potential targets of miR-223-3p and lncRNA-MEG3 was indicated as a probable candidate with two miR-223-3p binding sites (Figure 5A). In dual luciferase reporter gene assays, wild-type lncRNA-MEG3 and mutant sequences on one or both of the two binding sites (Site 1, Site 2, or Site 1 with Site 2) were employed in a luciferase vector. miR-223-3p significantly reduced luciferase activity to 42% for wild type lncRNA-MEG3, and miR-223-3p suppressed luciferase activities to 62% and 89% for mutant lncRNA-MEG3 (Site 1 or Site 2, respectively). No shift of luciferase activities for miR-223-3p was observed on the double mutation (Site 1 with Site 2) of lncRNA-MEG3 (Figure 5B).

We compared the abundance of lncRNA-MEG3 than that of miR-223-3p to serve as a ceRNA. The levels of lncRNA-MEG3 and miR-223-3pwere calculated as 1002 and 1100 copies per cell, respectively (Figure 5C).

### 2.5. Overexpression of miR-223-3p Rescues the Inhibitory Effect of TTR on Neovascularization

**Figure 6:**
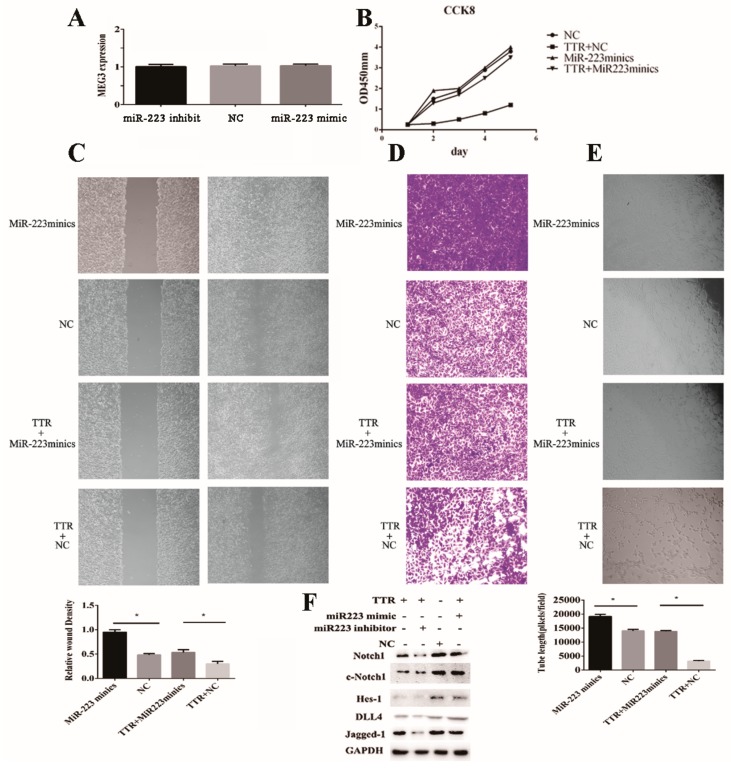
Overexpression of miR-223-3p blocks the inhibitory effect of TTR on neovascularization. (**A**) The expression of MEG3 was not affected by the level of miR-223-3p (repeated independently three times) (**B**) In CCK8 assay, the miR-223-3p mimic could promote the proliferation hRECs, while TTR could rescue partially rescue this trend (repeated independently three times) (**C**) In wound healing assay, the miR-223-3p mimic could increase the healing process, but TTR could repress the wound healing of hRECs (repeated independently three times; *, *p* < 0.05) (×100 magnification times). (**D**) In tranwell assay, miR-223-3p mimic could enhance the migration of hRECs, while TTR showed an inhibition function (repeated independently three times) (×100 magnification times). (**E**) In tube formation assay of hRECs, miR-223-3p mimic promoted vascularization; however, TTR repressed angiopoiesis (repeated independently three times; *, *p* < 0.05) (×100 magnification times). (**F**) The effects of miR-223-3p and TTR on Notch signal pathway was investigated using western blot analysis.

After the knockdown and overexpression of endogenous miR-223-3p, the level of lncRNA-MEG3 did not significantly fluctuate, which suggested miR-223-3p should be the downstream target of lncRNA-MEG3 (Figure 6A). It is noticeable that overexpression of miR-223-3p significantly promoted neovascularization, while the addition of TTR reversed this phenomenon (Figure 6B–E).

As Notch1 pathway was reported to be closely associated with neovascularization [36], western blot assays demonstrated that TTR inhibited the levels of Notch1, c-Notch1, Jagged-1, DLL4, and Hes-1, while miR-223-3p inhibitor or mimic enhanced or reversed this trend, respectively (Figure 6F).

### 2.6. TTR/LncRNA-MEG3/Axis Regulated Neovascularization in DR

**Figure 7:**
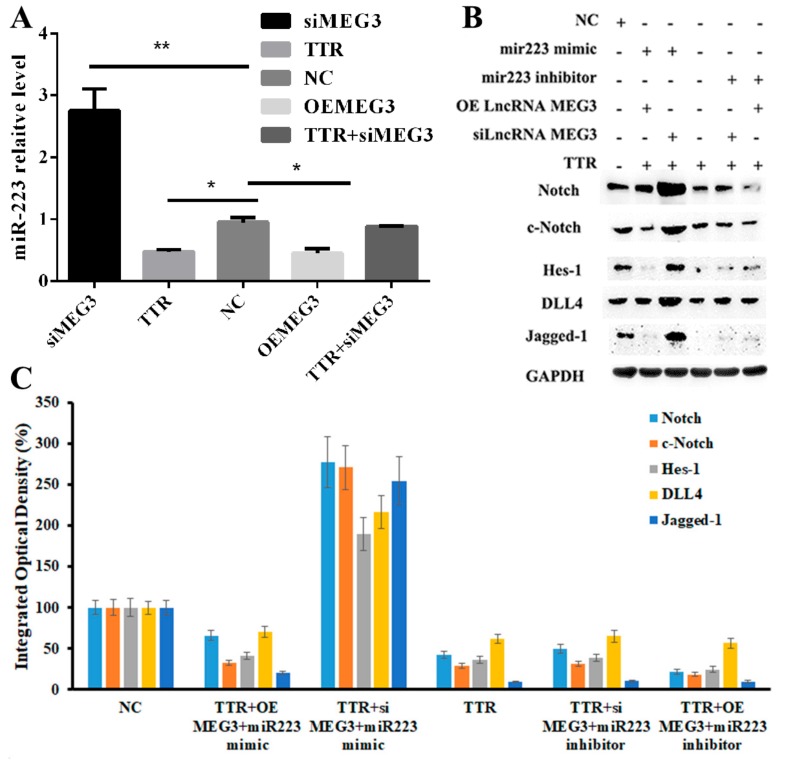
TTR/lncRNA MEG3/miR-223 network regulates retinal endothelial cell function in vitro. (**A**) In qRT-PCR analysis, TTR or overexpression or knockdown of MEG3 could significantly regulate the level of miR-223-3p (repeated independently three times; *, *p* < 0.05, **, *p* < 0.01). (**B**,**C**) Using western blot assay, miR-223-3p mimic could enhance the Notch pathway and then could further promote angiopoiesis; however, high abundance of TTR could reverse the function of miR-223-3p by regulating the level of lncRNA MEG3, which had been proved to be the ceRNA of miR-223-3p. The experiment was repeated independently three times.

To elucidate the role of the TTR/LncRNA-MEG3/miR-223-3p axis, it was shown that TTR could significantly reverse high level of miR 223-3p induced by MEG3 knockdown, while lncRNA-MEG3 overexpression reduced miR-223-3p levels, and it was in accordance with TTR. TTR with siMEG3 rescued the diminished miR-223-3p levels (Figure 7A). Western blot assays showed that TTR and lncRNA-MEG3 overexpression downregulated the levels of Notch1, c-Notch1, Jagged-1, DLL4, and Hes-1, while miR-223-3p mimic or inhibitor partially rescued or promoted the above phenomenon (Figure 7B,C). These results have suggested that TTR should inhibit the neovascularization process in DR by regulating theMEG3/miR-223-3p axis. 

## 3. Discussion

DR is associated with visual loss, and therefore a comprehensive understanding of the underlying mechanisms of DR might identify useful diagnostic or therapeutic targets [39]. Regarding some clinical investigations of ocular diseases, myopia is suggested to prevent patients from suffering DR [11,12]; therefore, in our previous work, we studied the relationship between the higher vitreous TTR level of high myopia patients [13] and the DR protection phenomenon. It is interesting that the serum and vitreous TTR levels in DR patients should be associated with DR progression [15], and in vitro, TTR has been proved to prevent the progression of DR [14], and repress angiogenesis by the Tie signaling pathway in hyperglycemia [16] or promote the apoptosis of hRECs by the GRP78-dependent pathway [17]. Additionally, TTR has also been proved to suppress the proliferation of hRECs by a TTR/STAT4/miR-223-3p/FBXW7 signaling pathway and could further affect the content of Notch1 [32]. However, these phenomenon and mechanism should only be a small part in the whole puzzle, and more investigations are necessary to reveal the detailed effects of TTR on the progression of DR.

In our previous work, although TTR could inhibit the proliferation and induce the apoptosis of hRECs in vitro [14,15,16,17,32], the effect of TTR on DR progression in vivo was unclear. Therefore, in this work, the DR mice model was induced with STZ, and after intravitreal injection of adeno-associated virus (AAV) vector containing TTR cDNA, the pathological progression of DR was partially reversed with overexpressed TTR (Figure 1). This suggested that TTR could repress the progression of DR both in vitro and in vivo. 

On one hand, to find out how could TTR affect miR-223-3p, immunoprecipitation (IP) was used to screen the direct target of TTR and total bind samples were applied for LTQ-MS analysis, while PABPC1 was identified with high abundance (data not shown). Furthermore, the directed interaction between TTR and PABPC1 was confirmed by co-IP (Figure 2A). 

On another hand, to search for the downstream factor of miR-223-3p was a challenge; as reported, lncRNA could regulate biological functions as a ceRNA or molecular “sponge” for other RNA molecules [40,41]. Several lncRNAs associated with ocular diseases and vascular progression were considered as potential candidates, including MEG3 [30,31]. In this work, the direct binding of lncRNA-MEG3 and miR-223 at two sites was proved in luciferase reporter assays (Figure 5); overexpression or knockdown of lncRNA-MEG3 could significantly decrease or enhance the level of miR-223-3p (Figure 7A), but miR-223-3p could not reveal the same effects on lncRNA-MEG3 (Figure 6A), which suggested that miR-223 should be the direct downstream target of lncRNA-MEG3. In vitro, addition of TTR could enhance the level of lncRNA-MEG3, and overexpression of lncRNA-MEG3 revealed the same repression function on the proliferation, migration, and tube formation properties of hRECs as TTR (Figure 3) in hRECs. In vivo, by intravitreal injection, lncRNA-MEG3 also showed the same protection function on the pathological progression of DR in the STZ-induced DR mice model (Figure 4). These results suggested that there might be a potential relationship between TTR and MEG3.

Additionally, according to previous reports, PABPC1 could bind the poly(A) tails of mRNAs and further regulate the stability and biofunction of lncRNAs [33,34,35], which indicated a potential relationship between PABPC1 and lncRNA-MEG3. Because lncRNA-MEG3 showed no significant effects on PABPC (Figure 2B), while the knockdown and overexpression of PABPC1 could significantly decrease or promote the level of lncRNA-MEG3 (Figure 2C), lncRNA-MEG3 should be the downstream target or PABPC1. 

**Figure 8:**
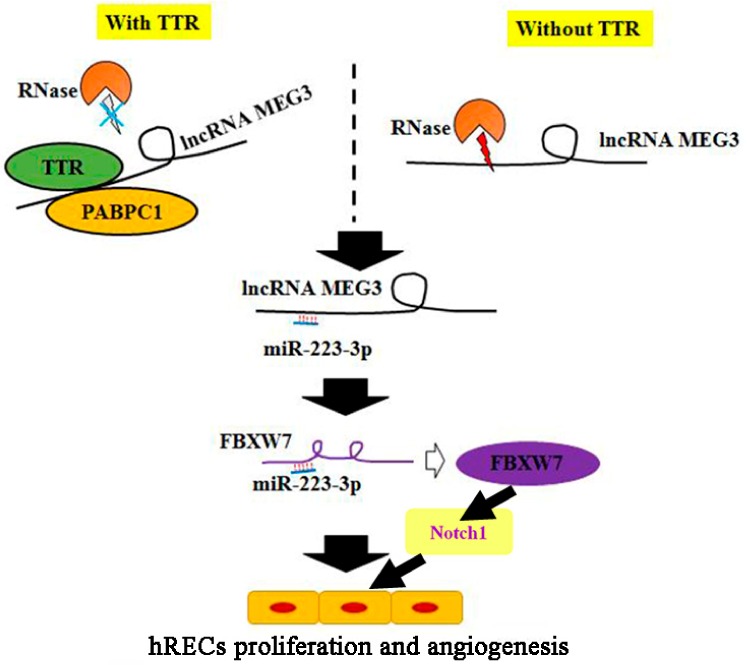
Schematic overview of the TTR/MEG3/miR-223-3p/FBXW7/Notch1 signaling axis.

Then, a TTR/PABPC1/lncRNA-MEG3/miR-223-3p signal axis was suggested by the results of this work. In our previous study, miR-223-3p has been proved to directly interact with 3’ UTR of FBXW7, and further affect the Notch1 pathway [32]. Combined with the results of this work, a more complete pathway was suggested: TTR could bind to PABPC1 and increase its content, PABPC1 could further bind and stabilize lncRNA-MEG3, which could decrease the level of miR-223 and further promote the level of FBXW7, and then the Notch1 pathway could be inhibited (Figure 8). 

## 4. Materials and Methods 

### 4.1. Ethics Statement

This study was certified by the Ethics Committee of Nanjing Medical University (2014-062 approved on February 26th, 2014; 2019-398 approved on February 25th, 2019). All animal experiments adhered to institutional guidelines for humane treatment of animals, the Principles of Laboratory Animal Care (National Institutes of Health, Bethesda, MD, USA), and the Association for Research in Vision and Ophthalmology (ARVO) Statement for the Use of Animals in Ophthalmic and Vision Research.

### 4.2. Proliferation Assay

hRECs were plated at a density of 3000 cells/100 μL medium in 96-well plates. After 24 h of incubation, the cell counting kit-8 (CCK8, Dojindo Laboratories, Kumamoto, Japan) was used to examine hREC proliferation [10,12].

### 4.3. Vascular Tube Formation Assay 

A basement membrane matrix (BD Biosciences, Franklin Lakes, NJ, USA) was used to detect tube formation. Matrix was loaded in a 48-well plate, and after drying for 3 min at 37 °C, 3 × 10^4^ hRECs were seeded in each well, followed by 6 h of incubation at 37 °C. Vascular tube formation of the hRECs was evaluated using an Olympus microscope (IX-73).

### 4.4. Cell Migration and Healing Assay

To assess cell migration, hRECs were seeded into upper wells and incubated 24 h, then hRECs were fixed on the inserts with 4% paraformaldehyde, stained with crystal violet, and counted under a light microscope. For healing assays, hRECs were incubated until confluence on a 6-well dish, then scratched with a sterile 10 μL pipette tip. After 24 h of incubation, wells were scanned with IncuCyte ZOOM (ESSEN BioScience, Ann Arbor, MI, USA), and relative wound density was evaluated.

### 4.5. qRT-PCR

Total RNA was extracted using an RNAiso Plus kit from Takara (Dalian, China). Total miRNAs were extracted and purified using a TaqMan® miRNA ABC Purification Kit from ThermoFisher Scientific (Carlsbad, CA, USA) (cel-miR-39-3p was added as spike-in as previously reported [20]). After cDNA of lncRNA-MEG3 was amplified with M-MLV reverse transcriptase (Invitrogen, Carlsbad, CA), qRT-PCR was carried out using SYBR Green Real-Time PCR Master Mixes (Takara, Dalian, China) (using GAPDH as internal control); to detect the levels of miR-223-3p in serum, the cDNAs were amplified and applied for qRT-PCR analysis using a mirVana™ qRT-PCR miRNA Detection Kit from Invitrogen (Carlsbad, CA, USA). The primers were designed using the OligoArchitect™ Online service (https://www.sigmaaldrich.com/china-mainland/zh/technical-documents/articles/biology/oligoarchitect-online.html) and miRprimer (https://sourceforge.net/projects/mirprimer/), respectively (Table 2) and synthesized by GenePharma (Shanghai, China).

### 4.6. Co-Immunoprecipitation (Co-IP) Assay and Western Blot Analysis

pcDNA3.1(+)-*PABPC1* and pEX-5-*TTR* plasmids were constructed by GenePharma (Shanghai, China), and then they were co-transfected into human embryonic kidney 293 (HEK293) cells using Lipofectamine 2000 (Invitrogen). Then, 1×10^6^ cells were lysed in pre-chilled RIPA buffer (incubated 2 h in ice water, 0 °C), and 20 mM PBS (1% octylphenoxypolyethoxyethanol (Nonidet P-40)/0.1% SDS/0.5% sodium deoxycholate/1 mM Na_3_VO_4_/5 mM NaF/10 mM sodium pyrophosphate/10 μg/mL aprotinin/5 μg/mL leupeptin/1mM PMSF) were added. The mixture was centrifuged for 10 min at 4 °C and 12000× *g*, pellets were depleted, and the proteins in the supernatant were loaded onto a Protein A column, using IgG as a blank. Protein samples were captured by Sepharose beads coupled with Protein A, incubated with approximately 2 μg of primary antibody for 12 h at 4 °C, washed with 20 mM PBS (0.5 M NaCl) five times, and boiled with 50 μL of 1× SDS loading buffer for 5 min. Captured proteins on Sepharose beads were then applied to SDS-PAGE and western blot assays.

### 4.7. Diabetes and DR Mouse Models Induced with Streptozotocin (STZ)

To establish models of diabetes and DR in mice, 50 μg/g of STZ were injected intraperitoneally daily for 5 d into 8-week old male C57BL/6 mice, using citrate buffer as a blank. The mice were considered to model diabetes once blood glucose surpassed 16.7 mmol/L. After intraperitoneal injection of 80 μg/g ketamine and 4 μg/g xylazine, mice were ventilated, and approximately 1.5 μL (1×10^12^ TU/mL) of adeno-associated virus containing TTR cDNA were intravitreally injected. For short-hairpin RNA (shRNA) or MEG3 shRNA adenovirus, mice were intravitreally injected every three weeks. 

There were two main animal treated assays designed (Evans blue leakage and Retina trypsing digestion assays), and after these treatments, mice could not be used repeatedly. Therefore, mice were divided into two groups for testing. Details of animal amounts have been exhibited in Table 1 and Figure 1 and Figure 4.

### 4.8. Retinal Imaging

After mice were anesthetized with 80 μg/g ketamine and 4 μg/g xylazine, Cyclometric (Alcon, Fort Worth, TX, USA) was used to dilate the pupils. A Micron IV image-guided OCT system (Phoenix Research Laboratories, Pleasanton, CA, USA) was then employed to detect spectral domain optical coherence tomography, guided by bright-field live fundus imaging.

### 4.9. Evans Blue (EB) Leak Assay

After mice were anesthetized with 80 μg/g ketamine and 4 μg/g xylazine, the mice were cannulated at the right jugular vein and the iliac artery, injected with heparinized saline, and injected with 45 μg/g EB into the jugular vein. Approximately 0.2 mL of blood were extracted from the mice after 2 h, then mice were perfused with PBS followed by 1% paraformaldehyde to the left ventricle. After the cornea, lens, and vitreous humor were depleted, the retina and sclera were fixed in 4% paraformaldehyde at 25 °C for 3 min. The retina was incubated in dimethylformamide at 78 °C for 12 h and centrifuged for 15 min at 12000× *g* before detection at A_620 nm_ (blue) and A_740 nm_ (background). 

### 4.10. Trypsin Digestion of Retina

After enucleation, mice eyeballs were fixed in 4% paraformaldehyde at 25 °C for 24 h. Retinas were extracted and digested with 3% trypsin at 37 °C for 3 h; the retinal vasculature was then washed, located on glass slides, dried, and stained with periodic acid-Schiff.

### 4.11. Luciferase Reporter Assays

Transient transfections were performed using the Lipofectamine2000 transfection system (Invitrogen) on human embryonic kidney 293 (HEK293) 50,000 cells plated in a 24-well plate. At 24 h after transfection, the cells were lysed in 100 µL of passive lysis buffer, and firefly and Renilla activity was determined with a luminometer using the Dual Luciferase Assay System (Promega, Madison, WI) on 20 µL of lysate following the manufacturer’s handbook. The pGL3-luc plasmids containing luciferase reporter gene and MEG3 binding sites or wild/mutated miR-223-3p mimic were ordered from GenePharma (Shanghai, China).

### 4.12. Statistical Analysis

Data are presented as the mean ± SD from at least 3–5 independent experiments. Statistical comparisons between two groups were made using two-tailed Student’s *t* tests. Differences among three or more groups were compared by one-way ANOVA followed by least significant difference post hoc tests (SPSS 17.0, Chicago, IL, USA). *p* < 0.05 was considered as statistically significant.

## 5. Conclusions

In summary, this work demonstrated that TTR might have a critical role in the progression of DR, that it directly binds PABPC1, and that it stabilizes lncRNA-MEG3. Additionally, lncRNA-MEG3 might inhibit DR neovascularization by affecting the Notch pathway, and the TTR/lncRNA-MEG3/miR-223-3p axis mediates anti-angiogenesis in DR. These results provide novel insights on the molecular pathogenesis of DR, and will inform future research into novel lncRNA-directed diagnostic and therapeutic targets for clinical prevention and therapy of DR. Still, the exact mechanism of PABPC1 regulation of lncRNA-MEG3 remains unknown and merits further research. The present findings provide a new perspective to understand the relationships between lncRNAs and their interactions with proteins.

## Figures and Tables

**Table 1:** Physiological parameters of non-diabetic and diabetic mice. STZ: streptozotocin; lncRNA: long non-coding RNA; TTR: transthyretin; shRNA: short hairpin RNA; MEG3: maternally expressed gene 3.

	Initial		3 Months		6 Months		Surviving Number	
	Body Weight (g)	Glucose (mmol/L)	Body Weight (g)	Glucose (mmol/L)	Body Weight (g)	Glucose (mmol/L)	Start	End
**Non-diabetic (Ctrl)**	25.2 ± 2.1	6.51 ± 0.52	45.5 ± 2.7	6.22 ± 0.31	65.3 ± 3.7	6.88 ± 0.55	12	12
**STZ**	25.1 ± 1.9	25.1 ± 1.2 *	32.1 ± 1.5 *	25.5 ± 1.5 *	39.5 ± 2.8 *	26.5 ± 0.56 *	12	6
**STZ + Vector**	27.9 ± 2.2	28.8 ± 3.2 *	35.4 ± 3.8 *	30.2 ± 2.7 *	44.6 ± 5.8 *	32.3 ± 3.5 *	12	7
**STZ + scr + TTR**	31.4 ± 4.3	27.4 ± 2.6 *	30.6 ± 1.9 *	33.4 ± 4.1 *	37.4 ± 4.4 *	32.2 ± 3.6 *	12	7
**STZ + TTR**	24.9 ± 2.3	26.5 ± 2.1 *	34.1 ± 1.3 *	26.5 ± 1.3 *	40.5 ± 3.2 *	28.3 ± 1.2 *	12	8
**STZ + lncRNA-MEG3**	25.6 ± 1.6	26.1 ± 1.4 *	35.6 ± 1.5 *	26.7 ± 1.2 *	41.2 ± 2.9 *	27.5 ± 1.5 *	12	7
**STZ + scr**	25.9 ± 1.3	27.1 ± 2.5 *	34.6 ± 1.2 *	26.8 ± 1.1 *	42.3 ± 2.5 *	29.5 ± 1.9 *	12	6
**STZ + lncRNA-MEG3 shRNA**	25.7 ± 2.5	26.3 ± 1.9 *	35.1 ± 2.3 *	26.5 ± 1.8 *	41.5 ± 2.4 *	30.3 ± 2.1 *	12	6
**STZ+ lncRNA-MEG3 shRNA + TTR**	29.6 ± 3.8	29.1 ± 4.2 *	33.8 ± 3.0 *	30.2 ± 4.7 *	42.4 ± 3.1 *	34.7 ± 4.5 *	12	8

All data are shown as mean ± SEM. The difference in physiological parameters between non-diabetic and diabetic mice was determined by repeated measures ANOVA at different time points. “*” indicates a significant difference compared with the Ctrl group. scr: scrambled short-hairpin RNA.

**Table 2:** Primers for qRT-PCR assay.

	Primers	
**miR-223-3p**	**Sense**	5′-CAGAAAGCCCAATTCCATCT-3′
	**Antisense**	5′-GGGCAAATGGATACCATACC-3′
**cel-miR-39-3p**	**Sense**	5′-GTCACCGGGTGTAAATCAG-3′
	**Antisense**	5′-GGTCCAGTTTTTTTTTTTTTTTCAAG-3′
**Lnc-MEG3**	**Sense**	5′-AGCGCTTCTGAAGACCAAAC-3′
	**Antisense**	5′-GAACACAAAGACACCCAGCA-3′
**GAPDH**	**Sense**	5′-GCACCGTCAAGGCTGAGAAC-3′
	**Antisense**	5′-TGGTGAAGACGCCAGTGGA-3′

## Abbreviations

MDPI	Multidisciplinary Digital Publishing Institute
DOAJ	Directory of open access journals
TLA	Three letter acronyms
LD	Linear dichroism
DR	Diabetic retinopathy
TTR	Transthyretin
LncRNA	Long non-coding RNA
MEG3	Maternally expressed gene 3
LncRNA-MEG3	Long non-coding RNA of maternally expressed gene 3
shRNA	Short hairpin RNA
miR-223-3p	Micro RNA-223-3p
PABPC1	Polyadenylate-binding protein cytoplasmic 1
hRECs	Human retinal microvascular endothelial cells
Co-IP	Co-immunoprecipitation
hRPECs	Human retinal pigment epithelial cells
GRP78	78-kDa glucose-regulated protein
ceRNA	Competing endogenous RNA
OCT	Optical coherence tomography
HEK293	Human embryonic kidney 293
silncRNA-MEG3	Small interfering lncRNA-MEG3
OE lncRNA-MEG3	Overexpression lncRNA-MEG3
